# BMI1, ATM and DDR

**DOI:** 10.18632/oncoscience.211

**Published:** 2015-08-21

**Authors:** Xiaozeng Lin, Yan Gu, Damu Tang

**Affiliations:** Division of Nephrology, Department of Medicine, McMaster University; Father Sean O'Sullivan Research Institute, The Hamilton Center for Kidney Research, St. Joseph's Hospital, Hamilton, Ontario, Canada

**Keywords:** BMI1, ATM, double-stranded DNA breaks, DNA damage response

The polycomb group protein BMI1 is a component of the polycomb repressive complex 1 (PRC1). Although lacking enzymatic activity, BMI1 makes an important contribution to the intrinsic E3 ubiquitin ligase activity of PRC1 via binding of the catalytic subunit RING2. PRC1 catalyzes mono-ubiquitination of histone H2A at lysine 119 (H2AK119Ub), a well-recognized epigenetic marker associated with gene silencing [1]. A well-studied locus suppressed by BMI1 is *ARF/INK4A*, which encodes two tumor suppressors p16INK4A and p14/p19ARF with well-demonstrated functions in activating the pRb and p53 pathway, respectively. This suppression thus plays a major role in BMI1-mediated maintenance of the self-renewal of hematopoietic stem cells (HSCs), neural stem cells (NSCs), stem cells of other tissues, and most importantly, cancer stem cells (CSC) [2]. Histone ubiquitination is also an essential aspect of DNA damage response (DDR). In agreement with this knowledge, recent studies reveal a role of BMI1 in DDR regulation. Specifically, through ubiquitination of H2A and γH2AX, BMI1 enhances homologous recombination (HR)-mediated repair of double-stranded breaks (DSBs) [3, 2], an activity that sustains cancer survival during genotoxic agents-based therapies, thereby contributing to BMI1's ability in promoting tumorigenesis.

BMI1 also regulates other aspects of DDR. In general, DDR is constituted of two major intimately-connected processes: DNA lesion repair and checkpoint activation. In accordance with this knowledge, we have recently reported a role of BMI1 in compromising DDR checkpoint activation via reducing ATM activation [4]. In response to etoposide-induced DSBs, overexpression of BMI1 decreases γH2AX, ATM S1981 phosphorylation (ATMpS1981), CHK2pT68 (an established ATM target), and G2/M arrest in MCF7 and DU145 cells; knockdown of BMI1 reverses all these events. While the effects on γH2AX suggest that BMI1 indirectly reduces ATM-mediated checkpoint activation through enhancing DSB repair, multiple lines of evidence demonstrate otherwise.

**Figure 1:**
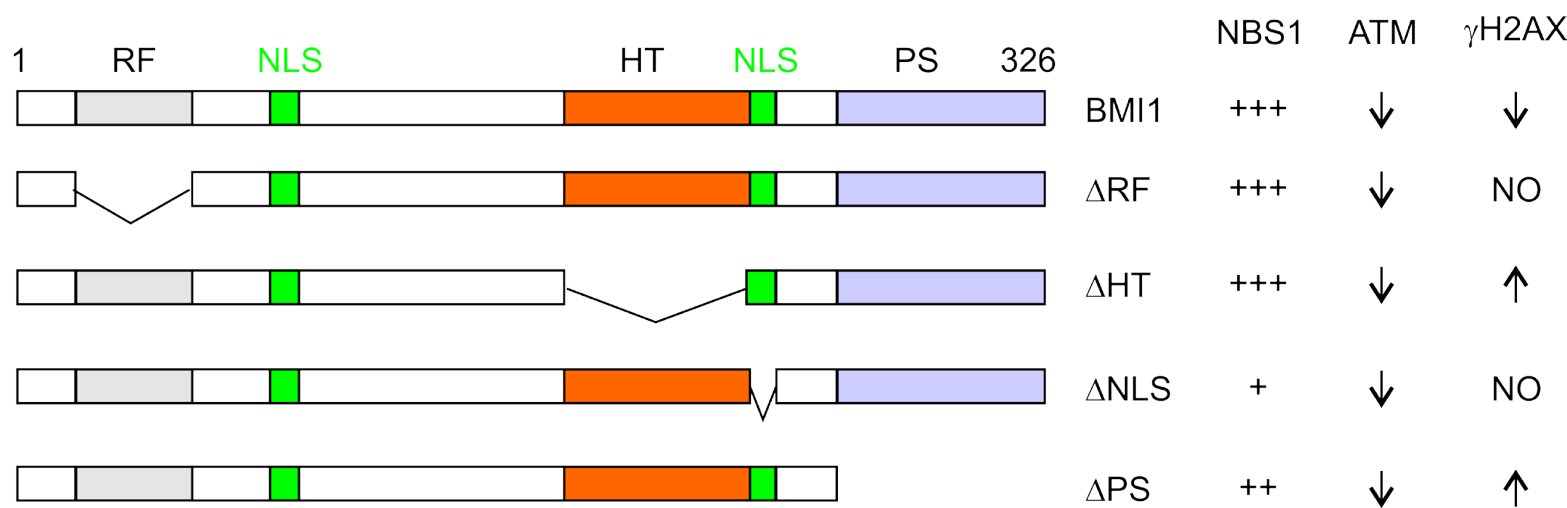
BMI1 reduces ATM activation independently of its activity in downregulating γH2AX in etoposide-elicited DDR BMI1 and the indicated deletion (Δ) mutants are shown for their activities in binding to NBS1 as well as affecting ATM activation and γH2AX levels. The last two events were determined in etoposide-treated MCF7 cells stably expressing the individual proteins. RF: ring finger; NLS: nuclear localization signals; HT: helix-turn-helix-turn-helix-turn; PS: proline/serine rich domain; number of + symbols indicating binding affinity; up and down arrows indicating decreases and increases of the indicated events; and NO: no effects.

DSB-elicited ATM activation requires binding of the MRN (MRE11-RAD50-NBS1) complex via a direct association with NBS1 [5]. BMI1 interacts with NBS1; this association alters the binding of NBS1 and ATM [4], which likely attenuates NBS1-mediated ATM activation. This possibility is supported by precipitation of the BMI1- NBS1-ATM complex in 293T cells co-transfected with BMI1 and ATM (our unpublished research). Individual deletions of a major nuclear localization site (NLS), HT (helix-turn-helix-turn-helix-turn), PS (proline/serine rich), or RF (ring finger) motif retains BMI1's ability in binding NBS1 and reducing ATMpS1981 and CHK2pT68 in response to etoposide treatment. However, these mutations affect BMI1's ability in decreasing γH2AX (Figure 1) [4], a surrogate marker of DSBs. Deletion of NLS (ΔNLS) renders BMI1 incapable of downregulating γH2AX in etoposide-treated MCF7 cells, which is likely result of reduced BMI1 nuclear localization. It was found that ΔHT and ΔPS elevated γH2AX (Figure 1), which can be attributable to their competition with endogenous BMI1 within PRC1, resulted in decreased E3 ubiquitin ligase activity and reducing BMI1's ability in enhancing HR-mediated DSB repair. These observations indicate BMI1 reducing ATM activation independently of its associated E3 ubiquitin ligase activity. This possibility is supported by BMI1ΔRF, a RING2-binding defective mutant that is not associated with the E3 ubiquitin ligase activity. However, BMI1ΔRF reduces ATM activation (Figure 1) [4] and confers resistance to etoposide-induced cytotoxicity in MCF7 cells (our unpublished research). Collectively, BMI1 is able to reduce ATM activation via binding of NBS1, independently of its-associated ubiquitin E3 ligase activity. This potentially brings up another question: is this BMI1 function also PRC1-independent?

This publication [4] opens a new avenue in pursuing BMI1-regulated DDR. BMI1 is commonly upregulated in a variety of cancer types and associates with cancer evolution. Genome instability lies in the heart of tumorigenesis and cancer progression. ATM plays a critical role in maintaining genome stability and its function is frequently reduced in cancer. Thus, there exists an intriguing possibility that BMI1 upregulation is an attribute to genome instability during tumorigenesis. This possibility can be addressed with translational research. Although BMI1 expression and ATM activation have been individually determined in all types of tumors, whether both events are associated remains unclear. Additionally, it will be informative to examine the impact of tissue specific BMI1ΔRF transgenic mice on tumorigenesis compared to the available tissue specific BMI1 transgenic mice [6]. These experiments are interesting, as BMI1ΔRF likely will not impact PRC1's E3 ubiquitin ligase activity.

BMI1 may also contribute to genome instability via other mechanisms. BMI1 was reported to bind nuclear PTEN [7], a PTEN population that maintains genome stability independent of its PIP3 phosphatase activity [8]. It can thus be envisaged that BMI1 inhibits nuclear PTEN function, thereby contributing to genome instability. This possibility fits well with the contrasting functions of the two proteins in regulating tumorigenesis.

BMI1 functions in two major aspects of DDR: HR-mediated DSB repair and checkpoint activation. This setting allows BMI1 to perform sophisticated activities in stimulating tumorigenesis. Under therapies with genotoxic agents, BMI1 promotes resistance via elevating DSB repair; when required to stimulate cancer progression, BMI1 contributes to genome instability.
